# Analysis of Neglected Tropical Disease Drug and Vaccine Development Pipelines to Predict Issuance of FDA Priority Review Vouchers over the Next Decade

**DOI:** 10.1371/journal.pntd.0001803

**Published:** 2012-10-25

**Authors:** Rianna Stefanakis, Andrew S. Robertson, Elizabeth L. Ponder, Melinda Moree

**Affiliations:** BIO Ventures for Global Health, San Francisco, California, United States of America; Duke University, United States of America

The need for new drugs and vaccines for neglected tropical diseases (NTDs) is widely accepted [1]. Yet, encouraging pharmaceutical and biotechnology company investment in developing these much-needed treatments remains a challenge due to a lack of a commercial market driving companies to pursue NTD projects [2]. To address this challenge, economists Ridley, Grabowski, and Moe at Duke University conceived of an incentive to encourage investment in the development of new drugs and vaccines for NTDs: the US Food and Drug Administration's (FDA) priority review voucher (PRV) program [3]. The program was signed into law on September 27, 2007 [4], and went into effect one year later.

Under the program, the FDA awards a voucher to the sponsor of a newly approved drug or vaccine that targets an NTD (such as cholera or dengue) or malaria and tuberculosis (TB). The voucher, which can be traded or sold, entitles the holder to a 6-month priority review for a future new drug application that would not otherwise qualify for priority review—potentially shaving between 4 and 12 months from the standard FDA review process [5].

Since the program's inception, only one PRV has been awarded, to Novartis Pharmaceuticals Co. for their 2009 approval of the antimalarial drug Coartem. Novartis used the voucher to accelerate the review of one of its own products, rather than selling it on the marketplace. Because a product resulting from a PRV has not yet been sold in the marketplace, the value remains uncertain. Early economic models estimated that the worth of a PRV could range from US$50 million to US$500 million, with an average value of US$322 million, and a variation in value based on the therapeutic area for which it is used [5], [6]. Part of predicting the value relies on the supply and demand of vouchers; that is, will the number of vouchers awarded be absorbed by the blockbuster products that are likely to be the intended recipients of benefit from accelerated review? The lack of understanding as to how many PRVs may be awarded in the future limits companies from predicting the potential value of a voucher that might be earned.

In the absence of a tangible example of a voucher's market value, companies, the FDA, policymakers, and other program stakeholders could benefit from examining NTD product pipelines, understanding when the next PRV(s) are expected to be issued, and ultimately quantifying the supply side of the PRV market. In addition, it is unclear to global health stakeholders whether companies are actively pursuing PRV-eligible products, and if they are, whether the PRV incentive has had an impact on their motivation [5], [6].

Here, we present an analysis of the drug and vaccine development pipeline to a) identify products that meet eligibility criteria to earn a PRV, and b) predict the number of PRVs that will be issued over the next 10 years. Of those products currently in clinical development, standard industry probabilities of success (POS) were applied to predict how many drugs and vaccines will ultimately earn regulatory approval, and therefore a PRV. Presumably, if stakeholders are armed with a supply forecast of the PRV market over the next decade, companies can conduct more informed calculations of value estimates, policymakers can assess whether the demand market for PRVs absorbs those vouchers being awarded, and the FDA can more accurately predict their expected workload increases when the PRVs are used.

## Identification of PRV-Eligible Drugs and Vaccines

To be eligible for a PRV, a drug or vaccine application must meet the criteria described in the *FDA Draft Guidance for Industry: Tropical Disease Priority Review Vouchers* [3]. Specifically, PRV-eligible products must be approved after the PRV program start date (September 27, 2007), must be for a human drug application submitted under section 505(b)(1) of the Act (for new chemical entities) or section 351 of the Public Health Service (PHS) Act (for biologics, or new molecular entities), and must contain no active ingredient (including any ester or salt of the active ingredient) that has been approved in any other application under section 505(b)(1) of the Act or section 351 of the PHS Act and, according to the FDA, “offer major advances in treatment, or provide treatment where no adequate therapy exists.”

**Figure 1:**
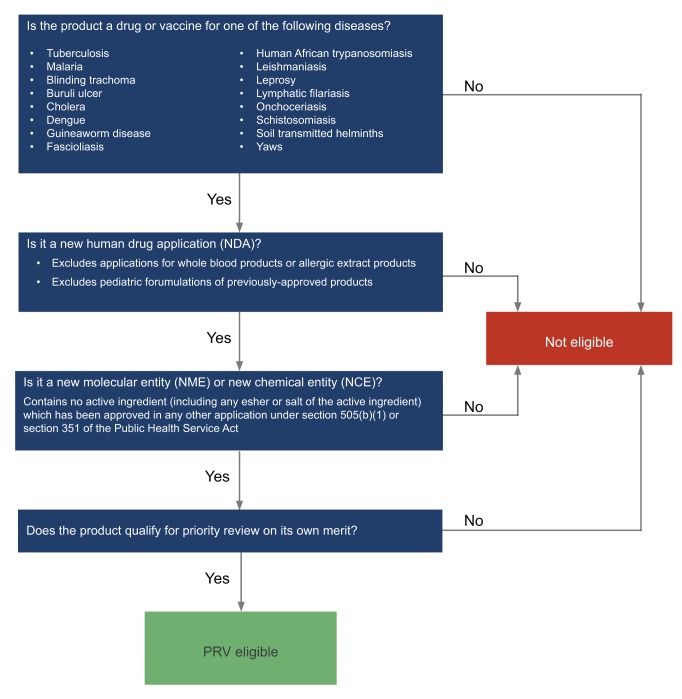
Eligibility decision tree for a US FDA priority review voucher. Criteria are based on the *FDA Draft Guidance for Industry: Tropical Disease Priority Review Vouchers.*

To identify drugs and vaccines in development that meet the PRV eligibility criteria, we examined the BIO Ventures for Global Health (BVGH) *Global Health Primer*, a unique dataset of NTD drug and vaccine development pipelines [7]. This unpublished dataset is the result of intensive reviews of the literature, examination of public pipeline information, and interviews with developers and disease-area experts. Across all disease pipelines, products that met PRV eligibility criteria were identified as “PRV-eligible,” with the disclaimer that this has not been vetted with the FDA. The decision tree used to select eligible products is based on the FDA Draft Guidance for Industry: Tropical Disease Priority Review Vouchers, and is outlined in Figure 1.

**Figure 2:**
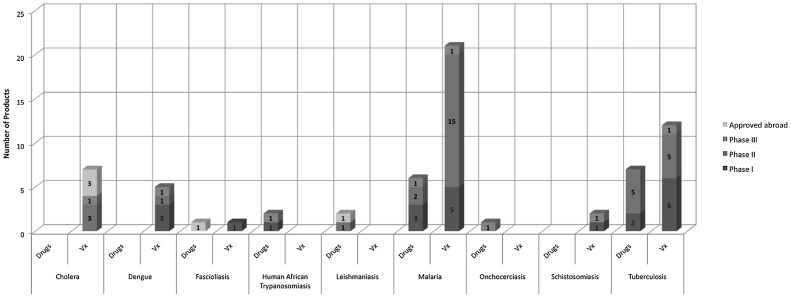
Number of PRV-eligible drugs and vaccines in the pipeline by phase of clinical development. Drugs and vaccines from the pre-clinical phase of development, through phases I, II, and III were evaluated for PRV eligibility (Figure 1). A total of 17 drugs in clinical development could earn a PRV, if approved by the FDA; two more are already approved abroad and would be eligible to earn a voucher if submitted to the FDA (six in phase I; eight in phase II; three in phase III). Forty-five vaccines are currently undergoing clinical development (16 in phase I; 24 in phase II; five in phase III); three additional vaccines are already approved abroad and would be eligible to earn a PRV if submitted to the FDA. The analysis excluded programs that are “on hold”.

Drugs and vaccines from the preclinical phase of development, through phases I, II, and III, were evaluated. Also, under the current statute, products that are approved abroad—but have not yet received FDA approval—and meet the eligibility criteria, could also earn a PRV if an application was submitted to the FDA. Therefore, we included products approved abroad that have not undergone review by the US FDA in our analysis (Figure 2).

Our analysis indicates a total of 17 drugs in clinical development could earn a PRV, if approved by the FDA; two more are already approved abroad and would be eligible to earn a voucher if submitted to the FDA (six in phase I; eight in phase II; three in phase III). For vaccines, we identified total of 45 currently undergoing clinical development (16 in phase I; 24 in phase II; five in phase III); three additional vaccines are already approved abroad and would be eligible to earn a PRV if submitted to the FDA (Figure 2).

## Applying Attrition Rates and Development Timelines to Predict Drug and Vaccine Success

Drug and vaccine POS were compiled from the most cited literature and industry reports. Separate analyses were conducted for drugs and vaccines, given the variation in POS for each product category. Selection of the appropriate POS percentages followed the PRV eligibility criteria. For instance, the ideal POS number would be for infectious diseases, and for new molecular entities and new chemical entities, and products that undergo “priority” versus “standard” review. Although some specific POS numbers have been calculated for specific diseases, such as TB, to our knowledge, no literature review or industry report has produced a study so highly tailored to these criteria [8]. Table 1 summarizes the range of estimates and the POS figures examined for this analysis [9]–[12]. The POS figures used for the analysis are summarized in the far right column. These represent the POS figures that most accurately encapsulate the PRV eligibility criteria, and therefore are the best predictors for the identified NTD products. These calculations also assume that each product that is approved by FDA and meets the PRV eligibility criteria (Figure 1) will receive a PRV.

**Table 1:** Average probability of success (POS) rates from various sources.

	Parexel Data, BCG Analysis (2008)		Kola & Landis (2004), *Nat Rev Drug Discov* a		Struck, *Nat Biotechnol* (1996)		Adams & Brantner, *Health Econ* (2010)		Rates Used for This Analysis	
	Drugs	Vx	Drugs	Vx	Drugs	Vx	Drugs	Vx	Drugs	Vx
Ph I – Ph II	0.77	-	0.65	-	0.88	0.72	0.75	-	0.77	0.72
Ph II – Ph III	0.68	-	0.4	-	0.86	0.79	0.48	-	0.68	0.62
Ph III – Registration	0.76	-	0.65	-	0.93	0.71	0.71	-	0.76	0.71
Registration-launch			0.95	-	1.00	0.96				

Values represent the probability of success (POS) that a product will move to the next stage of development.

a Kola and Landis rates represent those for the infectious disease therapeutic area.

Table 2 resulted from a review of the literature and industry reports that document average timelines for drug and vaccine development. Each clinical development phase involves certain studies that can take from 2 to 4 years, on average, to complete. The literature review produced three seminal studies that cite average development timelines based on historical data. Table 2 highlights the time intervals used for this analysis [13]–[16].

**Table 2:** Average timelines for development from various sources (in months).

	DiMasi et al. (2003)		PATH, MVI (2004)		Struck, *Nat Biotechnol* (1996)		Adams & Brantner, *Health Econ* (2010)		Rates Used for This Analysis	
	Drugs	Vx	Drugs	Vx	Drugs	Vx	Drugs	Vx	Drugs	Vx
Ph I – Ph II	21.6	-	-	24	21.6	24	16.58	-	21.6	24
Ph II – Ph III	25.7	-	-	36	26.4	21.6	30.65	-	25.7	36
Ph III – Registration	30.5	-	-	48	48	30	27.15	-	30.5	48
Registration - launch			-	-	19.2	15.6	-	-		

Values represent the average number of months it takes to complete a phase of clinical development.

MVI, Malaria Vaccine Initiative; PATH, Program for Appropriate Technology in Health.

Drug and vaccine clinical development timelines are long and costly, in addition to the time and cost of research necessary to reach that point. The overall average time for a drug to get through clinical trials is 6 to 11 years, plus an additional 0.6 to 2 years to receive regulatory approval through FDA [13], [15]. For vaccines, clinical development usually takes 6–8 years, with registration taking 12–18 months (Table 2) [15]. For this analysis, a 6-month priority review timeline was applied for the registration process, given that each product meets these criteria. This yields an average of 7.5 years for vaccine clinical development and approval.

## Estimated New Vouchers 2011–2020

Our analysis suggests a total of 17 drugs in clinical development that could earn a PRV; two more are already approved abroad and would be eligible to earn a voucher if submitted to the FDA (six in phase I; eight in phase II; three in phase III) (Figure 2). Based on POS, the analysis indicates that approximately ten PRVs could be awarded over the next 10 years.

**Figure 3:**
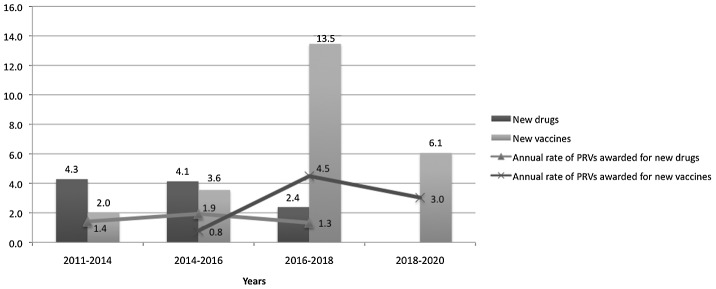
Estimated approvals of FDA drugs and vaccines for NTDs. Prediction model used to calculate the total number of expected PRVs, as well as the average annual rate of how many PRVs will be awarded. The model assumes a 100% success rate for approval by FDA of products already approved abroad. (The analysis adjusted for situations where one product approval would deem another ineligible. For example, SM14, a recombinant/purified protein vaccine, is currently under development for both schistosomiasis and fascioliasis. If it were to be approved for schistosomiasis, it would make approval for the indication of use against fascioliasis ineligible. Therefore, SM14 was counted as one product for the purposes of predicting the number of PRVs.) Similarly, the model assumes each individual product in development will yield one product. For malaria vaccines, for example, this may not be the case as many believe that these parts would be combined into one product before licensure. See study limitations for further explanation.

Applying the attrition rates for each phase, we predict that approximately two to three (actual statistic, 2.4) drugs will ultimately succeed that are currently in phase I (Figure 3). Of those products currently in phase II, approximately four (actual statistic, 4.1) are expected to succeed; and of those in phase III, approximately two (actual statistic, 2.3) are expected to succeed. The model assumes a 100% success rate for approval by the FDA of products already approved abroad. This adds two more drugs to the total of drugs that could earn a PRV (Figure 3).

Theoretically, these products that are approved abroad could be submitted to the FDA for approval in the near future, given that they have already undergone a regulatory approval process abroad. For this reason, the two drugs approved abroad were grouped with the products currently in phase III to estimate that approximately four (actual statistic, 4.3) PRVs could be earned within the next 3 years (2011–2014). Using the timelines for development identified in Table 2, in the period 2014–2016 we expect to see approximately four (actual statistic, 4.1) PRVs awarded for new drugs. From 2016 to 2018, those products currently in phase I that have a high probability of success will have reached the necessary endpoints for FDA approval; this could yield about two to three (actual statistic, 2.4) PRVs awarded (Figure 3).

For vaccines, we identified a total of 45 products currently undergoing clinical development (16 in phase I; 24 in phase II; five in phase III); three additional vaccines are already approved abroad and would be eligible to earn a PRV if submitted to the FDA (Figure 2). Based on vaccine-specific POS success identified in Table 1, we estimate as many as 25 PRVs being awarded for new vaccines over the next 10 years (Figure 3). For many reasons, this could be an overestimate, especially because the novelty requirements to earn a PRV are particularly unclear for vaccines. The limitations section below provides further explanation.

If the vaccine-specific POS and development timelines are applied, the model indicates that two vaccines could earn FDA approval and therefore a PRV within the next 3 years (2011–2014). These are the two vaccines that are currently approved abroad and could theoretically earn FDA approval if submitted in the near future. Subsequent analysis focused on vaccines currently in phase III that are likely to earn approval between 2014 and 2016. These calculations suggest 3.6 PRVs awarded during this period. From 2016 to 2018, a larger number of vaccines in phase I and II are expected to earn approval. This could translate to 13–14 (actual statistic, 13.5) PRVs awarded, with another six (actual statistic, 6.1) in the years 2018–2020 (Figure 3). As with the analysis of drug pipelines, this analysis assumes that the three vaccines that are already approved abroad would have 100% POS for their approval by FDA.

In the aggregate, up to 35 PRVs may enter the market over the next decade. An examination of the rate of PRVs awarded per year indicates a steady trend of about one to two PRVs per year for drugs (Figure 3). However, in the vaccines space, the first PRV to be awarded isn't expected until about 2015, after which there is a large increase in the expected rate of PRVs awarded annually from 2016 to 2018 (4.5 versus 0.8), with a reduction to about three PRVs per year between the years 2018–2020 (Figure 3). The discussion section below addresses this trend discrepancy between drugs and vaccines.

## Products Expected to Seek FDA Approval in the Next Five Years

The near-term implications of this analysis are most evident when we examine products that are in the final phases of clinical development, and therefore closest to seeking FDA approval and receiving a PRV. The products that could be submitted to the FDA for approval, versus other international regulatory agencies, are summarized in Table 3. The results suggest the next PRV will be awarded to the company that submits a new human drug application to the FDA for one of these products, should it be approved. Note that our estimates are the result of press release findings [17]–[19]. The FDA does not take public positions on the likelihood of approval and these predictions are based solely on typical times for phases of development and POS.

**Table 3:** NTD products expected to seek FDA approval in the next 5 years.^a^

Product Name	Product Type	Disease	Developer(s)	Estimated Approval Date	Comments
Bedaquiline (TMC207)	Drug	Tuberculosis (multi-drug resistant)	Janssen/Tibotec, TB Alliance	∼2013	NDA has been submitted to FDA for approval and priority review status has been granted. [18]
Moxidectin	Drug	Onchocerciasis	Pfizer, WHO	∼2013–2014	This product is approved abroad. The estimated approval date is based on assumption that a phase III trial begins in 2011 for FDA submission.
ChimeriVax	Vaccine	Dengue	Sanofi Pasteur	∼2015–2016	Currently in phase III trials abroad.
PXVX-0200	Vaccine	Cholera	PaxVax	∼2015–2016	Product is approved abroad. The FDA accepted PaxVax's IND application and the company expects to being phase III trials later this year. [19]
RTS,S/AS01	Vaccine	Malaria	GSK, MMV	∼2015 [17]	

a Except for bedaquiline and PXVX-0200, it is unknown whether all developers plan to submit these products for approval from FDA. These are estimates based on the individual product meeting eligibility criteria. These predictions are not supported by any statements made by the FDA.

GSK, GlaxoSmithKline; IND, investigational new drug; MMV, Medicines for Malaria Venture; NDA, new drug application.

## Limitations

There are several potential limitations to this estimate. For one, many of the vaccines incorporate new technologies that regulatory bodies such as the FDA have not yet encountered. For instance, no DNA or viral vector vaccines are on market for humans [20]. Both malaria and TB have several products in the pipeline that fall under these categories. Therefore, the model is limited by uncertainty around what product the FDA will deem eligible to earn a voucher. An example would be vaccines being developed in prime-boost combinations: if the “prime” is approved, are new “boost” vaccines eligible? Or if they are only approved in the context of the “prime” vaccine, is this no longer a “novel” product? Unlike a drug combination, both the prime and the boost vaccines will be separately packaged but only approved to be used together.

Further, the analysis assumes each individual product in clinical development will yield one product. Some vaccines will be combined after being developed separately. If these vaccines are combined in a single shot building on an individual product, the presumption is that they are not eligible to earn a PRV. This would be similar for drug combinations too, so further clarity is needed from the FDA regarding the eligibility of combination products. Similarly, it must be considered that once a product is approved, such as a new vaccine for malaria, it is unclear if all of the products behind that one in the pipeline will continue to be developed and/or if they will seek FDA approval. The attrition rate for second generation products may be higher if they are not significantly better than first generation products. Consideration of these caveats is especially important when examining the results for PRVs from vaccines that are expected to reach approval between 2016 and 2018 (4.5 versus 0.8), as these predictions may be higher than the actual yield.

In addition, there are limitations to the attrition rates applied in this analysis. These include [10]:

- Biologicals have a higher rate of success from first-in-man to launch—approximately 24%;
- Licensing-in compounds has a consistently higher probability of success in most studies, at approximately 24%;
- Companies with research and development (R&D) budgets of less than US$400 million also have higher success rates of approximately 18% [10].

Our analysis did not take these considerations into account. Future studies could take the analysis one step further by dissecting each individual pipeline product and applying a product-specific attrition rate to yield a more accurate prediction of the number of PRVs that can be expected.

This analysis uses mean times for clinical development that are not specific to NTDs. In the US, the mean time for clinical development during the 1990s was 8.8 years for neglected diseases, compared with 5.4 years for other indications [21]. The comparatively poor performance is attributable to the low market viability of these compounds, and hence suboptimum funding and resulting delays in development compared with potentially more profitable projects [22].

A further limitation is our assumption that companies with a product approved abroad would be willing to submit an approval package to the US FDA. Several factors contribute to a company's decision to submit a drug or vaccine for approval through the US FDA; for example, the size and financial status of the company, the headquarters location, and the company's familiarity with the FDA process and data requirements. Yet since Coartem serves as the first example of a PRV award, despite its widespread use and regulatory approval by over 85 agencies worldwide, we believe this assumption was important given that the current legislation allows for this scenario.

Finally, the list of NTDs designated by the World Health Organization (WHO) was recently updated and expanded. If the FDA were to use its authority to change the list of PRV-eligible diseases, some of the products that are in development for disease currently on the list may no longer be eligible. Updating the PRV-eligible list could also make eligible other products not currently included in this analysis, such as those products in development for Chagas disease. Clarification from the FDA as to the eligibility of these products is needed. This is a policy area that can be addressed by the regulatory agency itself.

## The Future of the PRV Program

For the PRV program to succeed, it must demonstrate that sponsors are willing to spend resources to accelerate the review of drugs with potentially high market value by using a voucher. To motivate development of drugs for neglected diseases, the expected net present value of the PRV must exceed half of the R&D costs to develop the drug, because the other half of the R&D costs would be covered by the Orphan Drug Act tax credits [5]. This analysis offers stakeholders a baseline for evaluating the program's potential impact, and supports the FDA by predicting how many PRVs it may be awarding, and therefore how many will eventually be used to expedite the review of other products.

Although only one company has received a PRV to date, the findings indicate that significant growth is forthcoming as the program matures. This is especially true for the vaccine surge that is suggested by BVGH *Global Health Primer* pipeline data that project up to 13.5 vaccine approvals for the period from 2016 to 2018 (Figure 3). This result seems high, but a recent report by BVGH examined the vaccine landscape for NTDs and found that malaria and TB alone account for a large number of these products, as malaria currently has 19 vaccines in clinical development, and TB closely follows with 11 [20]. Although the analysis we present applies the most appropriate POS numbers, the true yield of PRVs awarded will likely be lower than estimates given the challenging process of drug and vaccine development.

The FDA plays a critical role by administering the PRV program. A recent bill introduced in the US House and Senate, the Creating Hope Act of 2011, aims to fix many restrictions that are a concern to the private sector and outside the FDA's purview. This bill fleshes out the mechanics of the program in much greater detail than the original legislation, and addresses issues such as the current limit on voucher transferability and the rules around how to use and transfer a voucher. These fixes are what industry has identified as some barriers to engaging with the program [23]. The bill also proposes expanding the PRV program to include rare pediatric diseases. This would dramatically expand the initial scope of the PRV program. Additional analysis would be helpful in guiding policy because if the supply side of PRVs exceeds the demand side, the value of the PRVs could be seriously diminished.

Finally, this analysis can also serve as a starting place for subsequent analyses that could more completely forecast the market for PRVs going forward. Future studies should examine the number of blockbuster and other high value products coming down the pipeline that might benefit from using a PRV. Some experts predict that more than 20 innovative drugs with the potential for annual sales of US$1 billion or more each have strong odds of winning FDA approval over the next 3 years [24]. Several of these may earn priority review on their own merits, and therefore not need the PRV, but some may benefit from the program. It remains to be seen if the blockbuster pipelines are robust enough that the number of PRVs available will be significantly low in comparison, thereby driving up the value of obtaining a PRV to a blockbuster product sponsor.

Addressing gaps in the NTD pipeline was the impetus for developing the regulatory-based incentive that Professors Grabowski, Ridley, and Moe proposed in their 2006 *Health Affairs* article, which was quickly adopted into law. The timelines for product development are notoriously long, and the success of the PRV program won't be clarified until we see the preclinical and discovery phases filling up with more novel drugs and vaccines. Until then, this paper hopefully elucidates for stakeholders what is possible over the next 5–10 years for the program.
